# Diagnosis and clinical implication of collision gastric adenocarcinomas: a case report

**DOI:** 10.1186/s40792-022-01543-1

**Published:** 2022-10-07

**Authors:** Hiromitsu Imataki, Hideo Miyake, Hidemasa Nagai, Yuichiro Yoshioka, Norihiro Yuasa, Junichi Takamizawa, Ayami Kiriyama, Masahiko Fujino

**Affiliations:** 1 Department of Gastrointestinal Surgery, Japanese Red Cross Aichi Medical Center Nagoya Daiichi Hospital, 3-35 Michishita-Cho, Nakamura-Ku, Nagoya, 453-8511 Japan; 2 Department of Laboratory Medicine, Japanese Red Cross Aichi Medical Center Nagoya Daiichi Hospital, 3-35 Michishita-Cho, Nakamura-Ku, Nagoya, 453-8511 Japan; 3 Department of Pathology, Japanese Red Cross Aichi Medical Center Nagoya Daiichi Hospital, 3-35 Michishita-Cho, Nakamura-Ku, Nagoya, 453-8511 Japan

**Keywords:** Collision tumor, Collision adenocarcinoma, Multiple gastric cancer, Gastric cancer

## Abstract

**Background:**

Collision tumors are a subtype of simultaneous tumors wherein two unrelated tumors collide or infiltrate each other. Collision gastric adenocarcinomas (CGA) are rare and difficult to diagnose, and their clinical implications remain unclear. Herein, we aimed to reveal diagnostic methods for CGA and provide insight into its implications.

**Case presentation:**

Among 1041 cases of gastric cancers (GCs) resected between 2008 and 2018, we included cases of confirmed CGA. Patients’ backgrounds, preoperative endoscopy findings, macroscopic imaging findings, and histopathology findings [including immunostaining for CK 7, MUC2, and mismatch repair (MMR) proteins] were investigated. The incidence of CGA was 0.5%: 5 of 81 cases having simultaneous multiple GCs. Tumors were mainly in the distal stomach. The CGA in two cases was between early cancers, in two cases was between early and advanced cancers, and in one case was between advanced cancers. There were three cases of collision between differentiated and undifferentiated types and two cases between differentiated types. Immunostaining with CK7 and MUC2 was useful for diagnosing collision tumor when the histology was similar to each other. Among ten GCs comprising CGA, nine tumors (90%) exhibited deficient MMR proteins, suggesting high microsatellite instability (MSI).

**Conclusions:**

CGA is rare and usually found in the distal stomach. Close observation of shape, optimal dissection, and detailed pathological examination, including immunostaining, facilitated diagnosis. CGAs may have high MSI potential.

**Supplementary Information:**

The online version contains supplementary material available at 10.1186/s40792-022-01543-1.

## Background

Collision tumors are a subtype of simultaneous multiple tumors, wherein two independent tumors collide with or partially infiltrate each other, with clear borders and without the histological transition of one tumor to another [1]. Collision tumors are rare and usually found during pathological examination of surgically excised specimens. They can be encountered in many organs, including the brain, lung, esophagogastric junction, liver, and uterus [1–5]; however, there are limited reports and only small case series that describe collision gastric adenocarcinomas (CGAs) [6–18]. A CGA, wherein two synchronous adenocarcinomas develop nearby, can have specific characteristics; however, the features and clinical implications remain to be clarified because it is rare and often difficult to diagnose.

This study aimed to reveal the methods for CGA diagnosis and provide insight into the clinical implications of CGAs.

## Case presentation

We reviewed a prospectively recorded database of patients with gastric cancers (GCs) who underwent gastrectomy at our department from January 2008 to December 2018. Of 1041 patients who underwent gastrectomy, 81 (7.8%) had multiple synchronous adenocarcinomas. Among them, we found five patients with CGAs (6.2%) by postoperative detailed macroscopic observation and histopathological examination. Herein, we defined CGAs as gastric adenocarcinomas that have collided with each other, with partial topographic separation and histologically clear borders and without a histological transition of one to another type of adenocarcinoma [1]. Suspected collision tumors involving adenocarcinomas with squamous differentiation (*n* = 3), neuroendocrine tumors (*n* = 3), and lymphomas (*n* = 1) were excluded. The patients’ medical histories, findings of preoperative endoscopy, macroscopic imaging of the resected specimens, and histopathology, including immunostaining with CK 7, MUC2, and mismatch repair (MMR) proteins, were investigated.

The study protocol was approved by the ethics committee of our hospital (Registration Number: 2020–318). All participants provided informed consent.

### Immunohistochemistry

Immunohistochemical staining for CK 7 and MUC2 was performed in two patients. Immunohistochemical staining for mismatch repair (MMR) proteins, including MLH1, MLH2, PMS2, and MLH6, was performed for the 14 multiple gastric adenocarcinomas in five patients with CGA. Deparaffinized 4-μm-thick sections from each paraffin block were exposed to 0.3% hydrogen peroxide for 15 min to block endogenous peroxidase activity. Antigen retrieval was performed by autoclaving sections in 10 mM citrate buffer (pH 6.0) for 10 min. Sections were stained with primary antibodies, including anti-MLH1 (ES05, 1:200 dilution; Dako, Glostrup, Denmark), anti-MSH2 (FE11, 1:200 dilution; Calbiochem, La Jolla, CA, USA), anti-PMS2 (A16-4, 1:200 dilution; Biocare Medical, Concord, CA, USA), and anti-MSH6 antibodies (SP93, 1:200 dilution; Spring Bioscience, Pleasanton, CA, USA). We used an automated stainer (Dako) and En Vision Detection System (Dako) according to the vendor’s protocol. Non-neoplastic epithelial and stromal cells served as internal positive controls. Tumors showing significantly reduced or the loss of expression of any MMR protein were deemed to be MMR-deficient. The immunohistochemical staining results were evaluated by two pathologists (AK and MF).

### Patient demographics and endoscopic findings

**Fig. 1:**
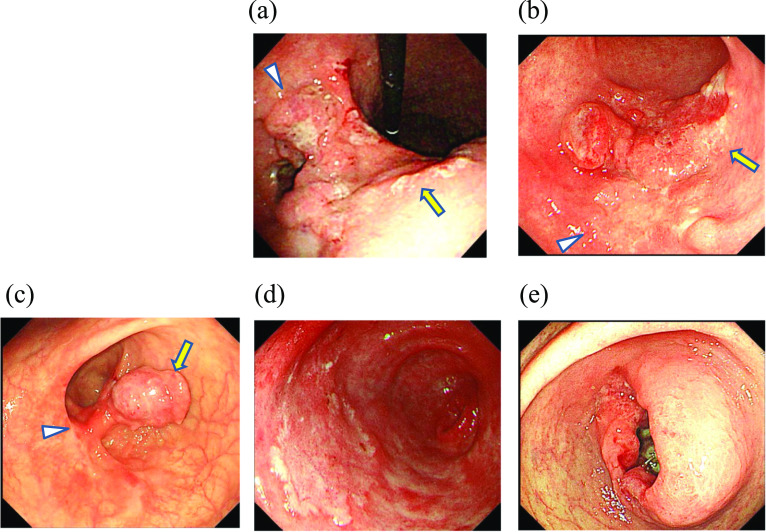
Endoscopic findings of five patients with collision gastric adenocarcinoma. **a** Case 1: a 66-year-old woman; two irregular ulcers with a marginal protrusion in the gastric angle (arrow, arrowhead). **b** Case 2: a 66-year-old woman; an irregular depressed lesion (arrowhead) and a distal adjacent, depressed lesion with marginal protrusion (arrow) of the gastric antrum. **c** Case 3: a 78-year-old man; a nodular elevated lesion (arrow) associated with a reddish depressed lesion (arrowhead) in the posterior wall of the gastric angle. **d** Case 4: an 81-year-old man; an irregular slight depressed lesion in the anterior wall of the gastric angle. **e** Case 5: a 75-year-old man; an elevated lesion with an irregular ulcer in the posterior wall of the gastric antrum

The demographics and characteristics of the five patients with CGA are shown in Table 1. The median age was 75 years (range, 66–81), and three patients were male. Endoscopic images of the five patients are shown in Fig. 1. A CGA was preoperatively suspected in one patient (Case 2), in whom an irregular, depressed lesion was adjacent to a distal, depressed lesion with marginal protrusion (Fig. 1b). Three distal and two total gastrectomies were performed.

**Table 1:** Summary of 5 cases of collision gastric adenocarcinoma

No.	Age	Sex	Surgery	Number of cancer	Collision cancer											Other cancer	
					Location	First cancer					Second cancer						
						Macroscopic type	Size (mm)	Depth of invasion	Histology	MMR	Macroscopic type	Size (mm)	Depth of invasion	Histology	MMR	Location	Macroscopic type, depth of invasion, histology
1	66	F	Distal Gx	2	LM	2	45	mp	por1	D	0–IIc	50	sm2	tub2	D		
2	66	F	Distal Gx	2	L	0–IIc	25	sm2	tub2	D	0–IIc	40	sm2	por1	D		
3	78	M	Total Gx	**5**	L	0–IIc	30	sm2	por2	P	1	40	mp	tub1	D	U M L	Type 3, ss, tub2 > por, pMMR 0-IIc, m, tub1, pMMR Type 3, mp, por, pMMR
4	81	M	Distal Gx	2	LM	0–IIc	20	m	tub1, CK7( +)	D	0–IIc	30	sm1	tub1, CK7(−)	D		
5	75	M	Total Gx	**3**	L	0–IIa + IIc	25	sm2	tub2, CK7( +), MUC2(−)	D	2	60	mp	tub2, CK7 focal ( +), MUC2 focal( +)	D	L	0-IIa, m, tub1, dMMR

*CK7* cytokeratin 7, *Gx* gastrectomy, *L* lower stomach, *M* middle stomach, *MMR* mismatch repair, *D* deficient, *P* proficient

### Macroscopic findings

**Fig. 2:**
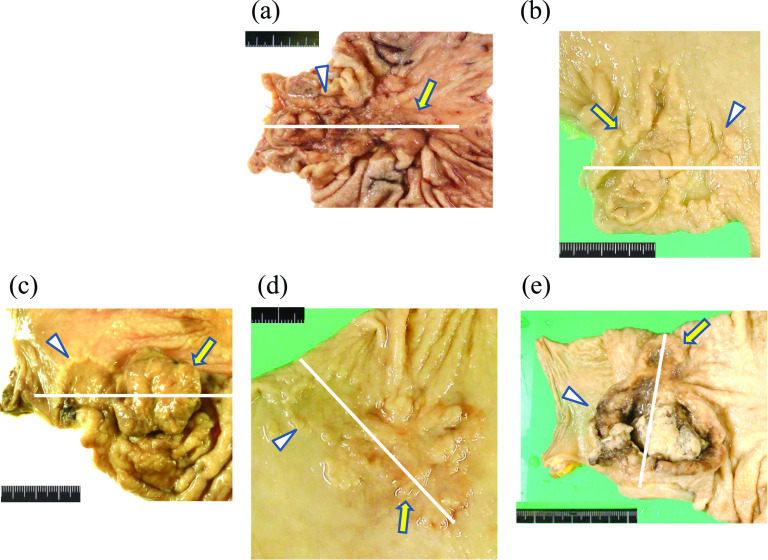
Images of formalin-fixed resected specimens divided according to the macroscopic findings of two adjacent lesions. The cutting lines were set perpendicular to the border of two adjacent lesions (arrows and arrowheads). **a** Case 1: an irregular, depressed lesion in the lesser curvature of the middle and lower stomach (arrow and arrowhead). **b** Case 2: a large irregular, depressed lesion with marginal protrusion (arrow) and a proximal adjacent depressed lesion (arrowhead) in the posterior wall of the lower stomach. **c** Case 3: a nodular elevated lesion (arrow) and a distal adjacent depressed lesion (arrowhead) in the posterior wall in the middle stomach. **d** Case 4: two adjacent irregular, depressed lesions (arrow and arrowhead) in the anterior wall of the middle stomach. **e** Case 5: a large, well-demarcated ulcer with marginal protrusion (arrowhead) and an adjacent small depressed lesion (arrow) in the lower stomach

Macroscopic images of fixed, resected specimens of the five patients are presented in Fig. 2. The number of GCs in each patient ranged from two to five. The location of the CGAs was mainly in the distal stomach. The macroscopic shapes were complex or bizarre due to the clear yet ambiguous borders of the multiple components. Formalin-fixed resected specimens were divided according to the macroscopic findings of two adjacent lesions; the cutting lines were set perpendicular to the border of the two adjacent lesions (Fig. 2).

### Histopathological findings

**Fig. 3:**
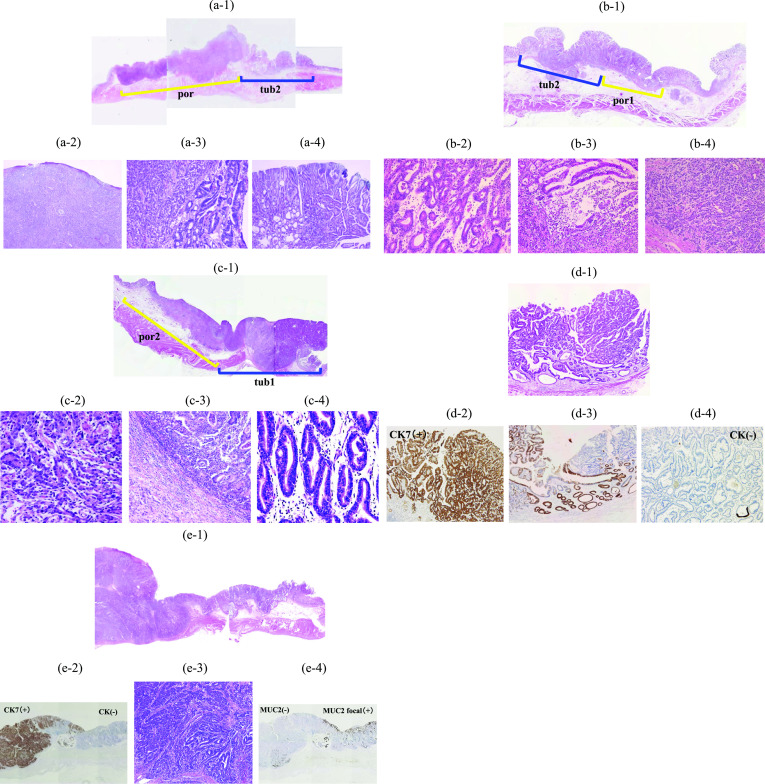
Histopathological findings of the resected specimen. **a-1–a-4** Case 1: a lesion with a poorly differentiated adenocarcinoma (por) collided with a lesion with a moderately differentiated tubular adenocarcinoma (tub2). **a-1** Loupe image (hematoxylin–eosin [HE]), **a-2** por (HE, × 40), **a-3** border of the two lesions (HE, × 100), **a-4** tub2 (HE, × 40) **b-1–b-4** Case 2: a lesion with tub2 collided with a lesion with poorly differentiated adenocarcinoma (solid type, por1). **b-1** Loupe image (HE), **b-2** tub2 (HE, × 100), **b-3** border of the two lesions (HE, × 40), **b-4** por1 (HE, × 100). **c-1–c-4** Case 3: a lesion with poorly differentiated adenocarcinoma (non-solid type, por2) collided with a lesion with well-differentiated tubular adenocarcinoma (tub1). **c-1** Loupe image (HE, **c-2** por2 (HE, × 100), **c-3** border of the two lesions (HE, × 40), (c-4) tub1 (HE, × 100). **d-1–d-4** Case 4: two tumors were similar tub2; however, they had a different positivity for CK 7. **d-1** Loupe image (HE), **d-2** CK positive (× 40), **d-3** border of the two lesions (× 40), **d-4** CK 7-negative (× 40). **e-1–e-4** Case 5: Two tumors were similar tub2; however, one tumor was CK 7-positive and MUC2-negative; and the other was CK 7-negative and MUC2 focally positive. **e-1** Loupe image (HE), **e-2** CK 7 staining (× 20), **e-3** border of the two lesions (HE, × 40), **e-4** MUC2 staining (× 20)

**Fig. 4:**
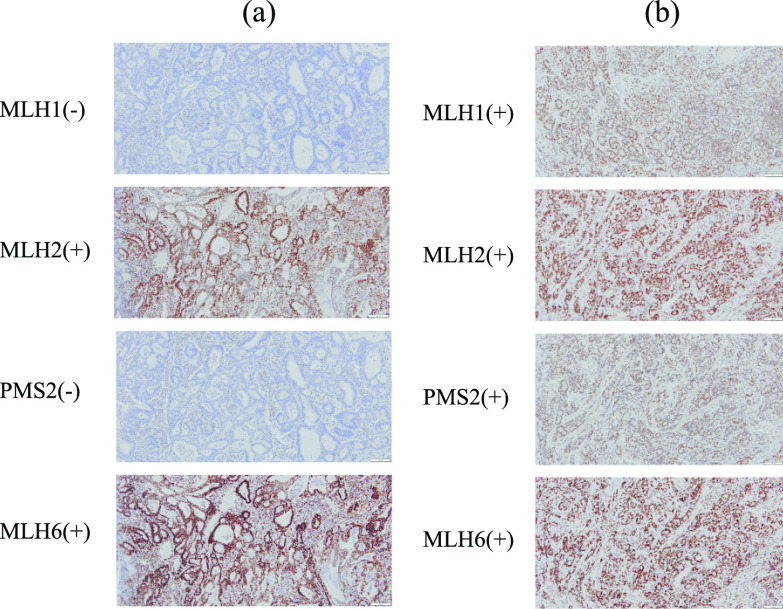
Immunohistochemical findings of mismatch repair (MMR) proteins in a representative colliding gastric adenocarcinoma (Case 3). **a** MLH1(−), MLH2( +), PMS2(−), and MLH6( +) indicating deficient MMR. **b** MLH1( +), MLH2( +), PMS2( +), and MLH6( +) indicating proficient MMR

The two lesions displayed different histopathologies, and the border was clear without transitional tissue in all five patients (Fig. 3). The two histopathologies were diagnosed as differentiated tubular and poorly differentiated adenocarcinomas by hematoxylin and eosin (HE) staining in three patients (Cases 1–3, Fig. 3a-1–4, b-1–4, c-1–4). Collision tumors were diagnosed by immunohistochemistry using CK 7 and MUC2 (Cases 4 and 5, Fig. 3d-1–4, e-1–4). In case 4, the two tumors were similar, well-differentiated tubular adenocarcinomas; however, immunostaining for CK 7 showed a difference in positivity (Fig. 3d-2–4). In case 5, both tumors were similar, moderately differentiated tubular adenocarcinomas; however, one tumor was CK 7-positive and MUC2-negative, and the other was CK 7-negative and focally MUC2-positive (Fig. 3e-2–4).

**Fig. 5:**
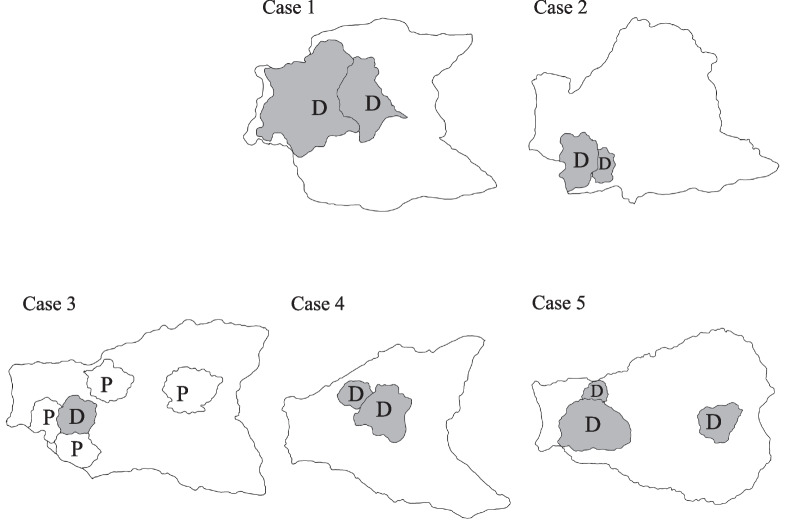
Schematic distribution and deficient/proficient mismatch repair protein (MMR) of 14 gastric cancers. *D* deficient MMR, *P* proficient MMR

We explored microsatellite instability (MSI) in the 14 GCs of the five study patients with retained MMR protein expression. Immunohistochemical findings of MMR proteins, including MLH1, MLH2, PMS2, and MLH6, in a representative case (Case 3) are shown in Fig. 4. Ten GCs showed deficient MMR proteins: MLH1(−), MLH2( +), PMS(−), and MLH6( +), while four GCs showed abundant MMR proteins: MLH1( +), MLH2( +), PMS( +) and MLH6( +). Of note, among ten GCs comprising CGA, nine (90%) exhibited deficient MMR proteins, suggesting high MSI (MSI-high) (Table 1). The schematic distribution of the deficient/abundant MMR of the 14 GCs is shown in Fig. 5.

None of the five patients experienced a relapse after gastrectomy; the median relapse-free survival was 32 months. One patient died of pancreatic cancer 32 months after gastrectomy.

## Discussion

This study showed that the incidence of CGA was 0.5% of the 1041 patients with surgically resected GC and 6.2% of the 81 patients with multiple synchronous adenocarcinomas. The collision tumors were identified by close macroscopic observation of their complex shapes, optimal division of the resected specimens, conventional HE staining, and immunostaining using CK 7 and MUC2. Among the ten collision tumors, nine exhibited deficient MMR proteins, suggesting high MSI.

Collision tumors are generally malignant tumors that originate primarily independently of each other at two separate sites and which later, in the course of their expansion, invade each other [19]. However, the diagnostic criteria for collision tumors have not been defined. In 1961, Dodge described a collision tumor as having separate tumor areas of two distinct histological patterns, which lack areas of transitional patterns or intermediate structures between the two types of tumors [1]. Later, Wanke and Spagnolo accepted some transitional patterns in the areas of collision [20, 21]. Because tumor collision may represent intratumor heterogeneity, we adopted Dodge’s definition, including the absence of transitional patterns and intermediate structures between the two types of tumors, to exclude tumors with suspected intratumor heterogeneity. Further, GCs with squamous differentiation, neuroendocrine tumors, and lymphomas were excluded.

Our extensive search of the English and Japanese literature (1996–2022) revealed 16 patients with CGAs according to the definition used in the present study (Additional file 1: Table S1) [6–18]. After the inclusion of our five patients, 21 cases were summarized in total. The median age of the patients was 70 years (interquartile range [IQR], 65–77 years), and 71% were men. The number of GCs in each patient ranged from two to five, and six patients (29%) had more than two adenocarcinomas. The location of the CGAs was mainly the distal stomach (n = 12), followed by the middle stomach (*n* = 7). Frequent macroscopic types of tumors comprising a collision tumor were type 2, 0–IIc, 0–I, and 0–IIa in 11, nine, six, and five cases, respectively. The median size of tumors comprising CGAs was 35 mm (IQR, 25–50 mm). More than half of the tumors were early GCs (mucosal and submucosal invasion in 12 and 13 tumors, respectively). Frequent histological types were differentiated tubular and poorly differentiated adenocarcinomas (22 and 14, respectively). Recently, cases that showed histopathological differences between the two components comprising a CGA by immunohistochemistry using EBER-ISH, TP53, MUC2, MUC5AC, and CK 7 have been reported [15–18].

CGAs are a subtype of multiple synchronous GC; therefore, several clinical characteristics overlap those of multiple GCs. Multiple synchronous GCs have been reported to account for 5–15% of all GC cases [22] and are associated with older age [23–26], being male [23, 27–29], the macroscopic type (elevated or depressed) [23, 30], the histologic type (differentiation) [24, 25, 31–33], the presence of intestinal mucin [25, 28], severe mucosal atrophy or intestinal metaplasia [25, 28, 34, 35], and submucosal ectopic gastric glands [35]. Multiple GCs are frequently associated with primary malignancies in other organs [33, 36–38], and the development of a metachronous GC after distal gastrectomy is clinically important [24, 39]. In addition, recent genetic studies indicate that MSI-high tumors are often (17–33%) observed in patients with multiple GCs [40–43]. We first investigated the MSI status in the CGAs and found a high rate (90%) of deficient MMR proteins, suggesting high MSI.

The Cancer Genome Atlas project classified GCs into four subtypes based on a comprehensive molecular evaluation: tumors positive for the Epstein–Barr virus, tumors with MSI, tumors with chromosomal instability, and genomically stable tumors. MSI-type tumors exhibit hypermethylation and elevated mutation rates and account for 5–22% of all GCs [44, 45]. Cho et al. hypothesized that the acquisition of an MMR deficiency occurs in the early stage of the gastric tumorigenesis associated with Lynch syndrome [46], which is caused by germline pathogenic variants in four MMR genes: MLH1, MSH2, PMS2, and MSH6 [47]. Meanwhile, sporadic MSI-high GCs may be related to hypermethylation of the MLH1 promoter [48]. Previous studies have reported a high prevalence (17–33%) of MSI-high in synchronous multiple GCs [43, 49]. MSI-high tumors have different clinicopathologic characteristics than MSI-low or MSI-stable tumors; MSI-high GCs are associated with older women, an intestinal-type (Lauren classification), middle and distal stomach locations, and fewer lymph node metastases [50–53]. In addition, Janjigian et al. reported that patients with MSI-high tumors suffered rapid disease progression after first-line standard cytotoxic therapy [54]. Treatment using monoclonal antibodies that target programmed death receptor-1 (PD-1) has shown promising results in patients with irresectable or metastatic MSI-high GC [55].

There are several hypotheses on the pathogeneses of collision tumors: (1) a carcinogenic stimulus on two neighboring mucosal regions resulting in the coexistence of two distinct neoplasms that later expand into each other and collide; (2) factors generated by an original tumor, such as gastrin’s trophic effect, granulocyte colony-stimulating factor, and immunosuppression, may induce the development of a neighboring second primary tumor (tumor-to-tumor carcinogenesis) [56–58]; (3) a common progenitor cell that grows contralaterally during cell division and afterward differentiates into two cell types that maintain their characteristics [59, 60]; and (4) malignant transformations and changes at the edge of an original tumor promote the development of a second distinct adjacent tumor [61]. A high rate of deficient MMRs was found in CGAs, suggesting that hypermethylation of the MLH1 promotor occurs in the adjacent gastric mucosa.

Our study has some limitations. CGA is a rare disease (0.5% of surgically resected GCs), so our study investigated just five patients. In addition, to reduce the possibility of intratumor heterogeneity, we adopted Dodge’s definition from 1961, which required only histopathological staining; therefore, it was easy to operate. Additional sequencing data may make it easier to confirm that the two tumors are distinct and originated independently.

Although rare, it is important to diagnose CGA accurately. If CGA is diagnosed with a single GC, several clinicopathological characteristics, including those of multiple GC and MSI, can be lost, affecting the choice of chemotherapy regimens, postoperative follow-up, and prognosis. Close macroscopic observation with the optimal cutting of the resected specimen and a detailed pathological examination, including immunostaining, can promote the accurate diagnosis of CGA.

## Conclusions

CGA is rare; however, its diagnosis is not difficult if close observation of the resected specimen and detailed pathological examinations are performed. CGAs have a significant potential for high MSI, and their correct diagnosis can affect the choice of chemotherapy regimens and postoperative follow-up.

## Supplementary Information

**Additional file 1: Table S1.** Reported cases of collision gastric adenocarcinoma.

## Data Availability

Data sharing does not apply to this article. The datasets supporting the conclusions of this article are included within the article and its additional file.

## Abbreviations

CGA: Collision gastric adenocarcinoma
GCs: Gastric cancers
HE: Hematoxylin and eosin
MMR: Mismatch repair
MSI: Microsatellite instability
